# Prevalence of workplace violence in Chinese obstetric nurses under the new situation and its correlation with violence prevention knowledge-attitude-practice and climate perception: a cross-sectional study

**DOI:** 10.1186/s12912-023-01637-7

**Published:** 2023-12-13

**Authors:** Shan Huang, Jinguo Zhai, Xiaoqin Lu, Yulian Liang, Qiumei Li, Hamza Saidi Lilenga

**Affiliations:** 1 School of Nursing, Southern Medical University, Guangzhou, 510515 China; 2 Nursing Department, Guangzhou University of Traditional Chinese Medicine Dongguan Hospital, No. 3, Dongcheng section, Songshan Lake Avenue, Dongguan City, Guangdong Province 523000 China; 3 Department of Obstetrics and Gynecology, Songshan Lake Central Hospital, No. 1, Xianglong Road, Dongguan City, Guangdong Province 523000 China; 4 School of Nursing, Southern Medical University, No.1023, South Shatai Road, Baiyun District, Guangzhou, Guangdong Province 510515 China

**Keywords:** Obstetric nurses, Midwives, Workplace Violence, Knowledge-attitude-practice, Violence climate perception

## Abstract

**Background:**

Workplace violence severely impacts individual nurses. With the three-child opening policy of China and the impact of the COVID-19 epidemic in the recently years, obstetric nurses face the double attack of heavy workload and staffing shortage. This study aimed to evaluate the current situation of workplace violence among Chinese obstetric nurses under the new situation, assess the level of violence prevention knowledge-attitude-practice and climate perception in Chinese obstetric nurses and explore the correlation between workplace violence and the level of violence prevention knowledge-attitude-practice and climate perception.

**Methods:**

A cross-sectional survey on the workplace violence for Chinese obstetric nurses was conducted from August to February 2023. All the questionnaires came from 10 provinces and 3 autonomous regions in China. The basic characteristics of obstetric nurse’s workplace violence, workplace violence prevention knowledge-attitude-practice scale and workplace violence climate perception scale were collected.

**Results:**

Totally, 522 Chinese obstetric nurses were included. 55.0% of obstetric nurses (287) had experienced workplace violence in the past 12 months, including verbal assault (40.4%), physical assault (34.1%), and sexual assault (31.0%). The overall level of obstetric nurses’ workplace violence prevention knowledge-attitude-practice and climate perception of workplace violence was low. Multiple linear regression analysis showed that the violence prevention knowledge dimension significantly influenced obstetric nurses’ workplace violence (B = -0.278, *P* < .001), attitude dimension (B = -0.241, *P* < .001), behavior dimension (B = -0.216, *P* < .001) and the violence climate perception’s organizational management (B = -0.177, *P* < .001), organizational training (B = -0.175, *P* < .001), organizational support (B = -0.143, *p* < .001) and the violence handling (B = -0.165, *P* < .001).

**Conclusion:**

The incidence of workplace violence among obstetric nurses in the new situation is high. However, the overall violence prevention knowledge-attitude-practice and climate perception levels are low. Therefore, nursing managers should take targeted measures according to the relevant influencing factors and the characteristics of obstetrics to improve the level of obstetric nurses’ violence prevention knowledge-attitude-practice and climate perception to reduce workplace violence among obstetric nurses.

**Supplementary Information:**

The online version contains supplementary material available at 10.1186/s12912-023-01637-7.

## Introduction

The World Health Organization (WHO) defines workplace violence (WPV) as verbal abuse, threats, and attacks on health personnel in the workplace, resulting in explicit or implicit harm to their safety, well-being, and health [1]. According to its manifestations, WPV has three forms, including physical aggression, verbal aggression, and sexual harassment [2]. Furthermore, according to the source of violence, WPV has four types, the most common of which are vertical violence and horizontal violence [3]. Workplace violence is a global social problem, especially in the medical industry, where its incidence is high. In recent years, workplace violence has seriously affected the physical and mental health of medical staff and has received extensive global attention [4]. The incidence of workplace violence at home and abroad ranges from 68.4 to 87.0% [5, 6]. A recent survey of 28 member States of the European Federation of Nurses (EFN) found that health professionals have an 80% higher risk of workplace violence [7]. Verbal violence was the most frequent type, amounting to 71.9% [8].

Nurses play a crucial role in clinical diagnosis and treatment, as they have extensive patient contact. However, this also puts them at a higher risk of conflicts with patients, leading to workplace violence (WPV). WPV negatively impacts nurses’ work quality, job satisfaction, and overall nursing standards [9, 10]. It also contributes to job burnout and intentions to quit, resulting in staff shortages and economic losses [10–14]. It’s important to note that WPV can even discourage midwifery students, leading to a decline in the quantity and quality of future midwifery professionals [15]. The Ockenden report on adverse clinical events in UK obstetrics highlighted staff shortages and a culture of bullying as factors contributing to these events [16]. Despite increased awareness and support, WPV continues to rise among nurses [5]. A recent survey by international health organizations revealed an increase in violence against healthcare workers worldwide since the COVID-19 pandemic in 2020 [16]. In China, the implementation of the two-child policy in 2016 and the three-child policy in 2021 resulted in a significant rise in elderly and high-risk obstetric deliveries. After the complete implementation of the two-child policy, the incidence of high-risk pregnancies increased by up to 20% [17]. This not only adds to the workload of obstetric nurses but also presents new challenges. Additionally, since the outbreak of COVID-19 in 2020, hospitals have faced increased support demands. The combination of increased workload and staffing shortages has significantly contributed to job burnout among obstetric nurses [18], which is a contributing factor to WPV [19]. Similarly, research by Capper et al. [10] found that WPV leads to the loss of obstetric nurses and affects the quality of maternal and child care, perpetuating a cycle of WPV in obstetric settings.

The knowledge, attitudes, and practices of nurses regarding workplace violence prevention are crucial in addressing this issue. It has been found that having a good level of violence prevention knowledge and maintaining a correct and positive attitude can positively influence the behavior of violence prevention [20]. Furthermore, nurses’ perceptions of the workplace violence climate, which refers to their views on the organizational policies and practices implemented by hospital management to control and eliminate workplace violence, play a significant role. A reasonable perception of the violence climate can reduce the negative impact of violence on nurses and effectively decrease its incidence [21, 22].

While there have been numerous studies on workplace violence among nurses in emergency departments, psychiatry, and geriatrics, there is a lack of research focusing specifically on Chinese obstetric nurses. A previous study highlighted that although obstetric medical staff face a lower risk of physical attacks compared to emergency departments, they experience similar levels of verbal violence, which is associated with job burnout [23]. Therefore, the present study aims to investigate workplace violence among obstetric nurses in the current context, including their levels of violence prevention knowledge, beliefs, and behaviors, as well as their perceptions of the violence climate. The study also aims to explore the impact of these factors on workplace violence experienced by obstetric nurses.

## Methods

### Design, setting, and participants

A cross-sectional online questionnaire survey was conducted among Chinese obstetric nurses by convenience sampling from August to February 2023. First, generate a link to an online electronic questionnaire platform, then publish the questionnaire and create a QR code. The researchers then sent a QR code with the help of Guangdong Nurses Association notice, explaining the purpose of the survey and the requirements of the respondents and inviting eligible obstetric nurses to participate in the study. Respondents responded to questionnaires by scanning the QR code on their mobile phones, eligible nurses knew that the study was voluntary and anonymous, and all participants provided informed consent. Finally, A red envelopes were sent to group users to express their gratitude for improving the answer rate of the questionnaire. The Inclusion criteria were as follows: (1) those who had obtained the nurse practitioner qualification certification; (2) those who had worked in obstetrics for more than one year; and (3) those who voluntarily participated in research on this subject. In addition, the exclusion criteria were: (1) those who left their posts for various reasons such as vacations and advanced studies; and (2) the nurses with evident mental illness. All participants’ information was kept strictly confidential.

### Sample size calculation

The calculation formula is N = *z*².*p*.(1-*p*)/*d*^2^. Among them, N is the required sample size, *p* is the probability of violence after a small-scale preliminary investigation, Researchers selected a 95% confidence interval for the *z* value, and the corresponding value is 1.96, *d* is the test level is 0.05 in this study. The study considered 10-20% as missing values. After pre-investigation, the incidence of WPV was 50%, and the sample size was at least 424.

### Measurements

#### Questionnaire on essential characteristics of obstetric nurses

The study team adopted a self-designed questionnaire on the essential characteristics of obstetric nurses, which included gender, age, educational background, marital status, whether only child, whether local, and other demographic characteristics, as well as obstetric nursing working years, professional title, and position, employment form, and other job characteristics.

#### Questionnaire on workplace violence among obstetric nurses

The questionnaire consists of two parts: frequency of WPV (3 items) and event situation (13 items). If respondents have yet to experience WPV, they will skip the WPV incident situation section. The questionnaire refers to the “Hospital Site Violence Questionnaire” [23]. The researchers then consulted obstetric specialists (the professional title was associate chief of nursing or above). Opinions of the experts guided the revision of entries. The modified questionnaire was first pre-surveyed with a small sample (10) in a tertiary care hospital, and the questionnaire was calibrated again after the pre-survey. After the formal survey, the questionnaire was no longer modified.

#### Workplace violence prevention knowledge-attitude-practice scale (Cronbach’s alpha = 0.969)

The scale refers to the “Nurse Horizontal Violence Prevention Knowledge-Attitude-Practice Scale” [20]. The revised scale contains three dimensions and 15 items. Each item was constructed based on a 5-point Likert scale (1 = strongly disapprove, 5 = strongly agree), with a score ranging from 15 to 75. The higher the score, the higher the level of violence prevention knowledge-attitude-practice. Cronbach’s α of the scale was 0.969, and Cronbach’s α values of the three dimensions ranged from 0.953 to 0.985.

#### Workplace violence climate perception scale (Cronbach’s alpha = 0.984)

The scale refers to the “Workplace Violence Climate Perception Scale” [23] designed by Huang et al. The revised scale contains four dimensions and 24 items. On a 5-point Likert scale (1 = strongly disapprove, 5 = strongly agree), the score ranges from 24 to 120. The higher the score, the higher the Workplace Violence climate perception level. The Cronbach’s α of the scale was 0.984, and Cronbach’s α values of the four dimensions ranged from 0.846 to 0.995.

### Statistical analysis

SPSS 26.0 was used to analyze the data. Descriptive statistical methods described the essential characteristics of obstetric nurses, workplace violence, violence prevention knowledge-attitude-practice, and climate perception. One-way ANOVA and Pearson correlation coefficients were used. Multiple linear regression was used as equations as control variables for significant variables identified in the one-way ANOVA. Significant variables identified in the Pearson correlation were used as independent variables to test the individual contribution of each potential independent variable to the dependent variable.

## Results

### Basic characteristics

Five hundred thirty-nine questionnaires were received, of which 522 were valid, with an effective rate of 96.85% (questionnaires with a completion time of fewer than 3 min or incomplete contents were invalid questionnaires). As shown in Table 1, most of the 522 obstetric nurses surveyed were in general public hospitals (62.8%) and tertiary hospitals (78.5%). Female participants accounted for 99.4%. 24.0% were unmarried, and approximately 74.3% were married. Most (76.2%) participants had a bachelor’s degree or above. Among the 522 obstetric nurses included, approximately 44.3% had junior titles, and 40.8% had intermediate titles. Most were in the 20–30 (32.2%) and 31–40 (51.2%) age groups. The working years were mainly concentrated in 6–10 years (28.7%) and 11–20 years (37.6%). Among the 522 obstetric nurses, 296 (56.7%) were midwives, 70.7% were general front-line nurses, and 310 (59.4%) were contract workers. Non-only child nurses accounted for a large proportion (90.4%), and nurses with prescription rights accounted for a small proportion (20.3%). There are significant differences of age, working years, professional title, and current marital status among obstetric nurses.

**Table 1:** Basic characteristics of obstetric nurses and univariate analysis of their relationship with workplace violence (*N* = 522)

Variables	Number (Percentage, %)	WPV (mean ± SD)	Statistical values	*p*
Gender			0.007^a^	0.934
Male	3(0.6)	3.00 ± null		
Female	519(99.4)	4.43 ± 1.80		
Age(years)			3.748^a^	0.024*
20–30	168(32.2)	4.13 ± 1.55		
31–40	267(51.2)	4.53 ± 1.85		
> 40	87(16.7)	4.63 ± 2.04		
Years of experience in obstetric care(years)			3.155^a^	0.014*
< 3	41(7.9)	3.66 ± 1.04		
3–5	61(11.7)	3.98 ± 1.27		
6–10	150(28.7)	4.49 ± 1.82		
11–20	196(37.6)	4.55 ± 1.90		
> 20	74(14.2)	4.72 ± 2.07		
Education			1.262^a^	0.287
Technical secondary school	5(1.0)	4.40 ± 2.07		
College degree	119(22.8)	4.17 ± 1.45		
Bachelor’s degree	389(74.5)	4.50 ± 1.89		
Master’s degree or above	9(1.7)	4.44 ± 2.13		
Professional title			3.103^a^	0.026*
Senior title	57(10.9)	4.56 ± 1.96		
Intermediate title	213(40.8)	4.53 ± 1.89		
Junior title	231(44.3)	4.35 ± 1.73		
uncertain	21(4.0)	3.62 ± 0.97		
Profession			0.63^a^	0.533
Midwifery	296(56.7)	4.51 ± 1.91		
Ward maternity nurse	207(39.7)	4.37 ± 1.69		
Assistant Nurse	19(3.6)	3.53 ± 0.84		
position			2.122^a^	0.121
Nurse	369(70.69)	4.35 ± 1.70		
Nurse group leader	88(16.86)	4.47 ± 1.95		
Head Nurse	65(12.45)	4.74 ± 2.12		
Marital status				
Single	125(24.0)	4.28 ± 1.79	9.065^a^	0.000**
Married	388(74.3)	4.47 ± 1.82		
Other	9(1.7)	4.11 ± 1.17		
Whether they grew up in a single child family				
Yes	50(9.6)	4.66 ± 1.92	3.023^a^	0.083
No	472(90.4)	4.39 ± 1.79		
Form of employment			2.556^a^	0.079
Formal worker	199(38.1)	4.37 ± 1.85		
A contract worker	310(59.4)	4.47 ± 1.79		
Temporary workers	13(2.5)	4.00 ± 1.35		
Whether local or no			1.2^a^	0.274
Yes	210(40.2)	4.53 ± 1.87		
No	312(59.8)	4.35 ± 1.76		
Whether you have prescriptive authority related to nursing work			0.769^a^	0.381
Yes	106(20.3)	4.30 ± 1.56		
No	416(79.7)	4.45 ± 1.86		
Nature of current hospital			2.032^a^	0.108
Public General Hospital	328(62.8)	4.46 ± 1.83		
Public specialist hospital	173(33.1)	4.37 ± 1.74		
Private General Hospital	20(3.8)	4.20 ± 1.91		
Private specialist hospital	1(0.2)	4.00 ± null		
The grade of the hospital			0.807^a^	0.447
First-class hospitals	33(6.3)	3.85 ± 1.37		
The secondary hospital	79(15.1)	4.41 ± 1.62		
Tertiary hospitals (Excluding Pre- mium Grade)	410(78.5)	4.47 ± 1.86		

Abbreviations: WPV, workplace violence; SD, standard deviation

Analysis of variance (ANOVA); p, statistical significance

*p < .05

**p < .01

### Workplace violence in obstetric nurses

Of the 522 obstetric nurses, approximately 55.0% (287) reported that they had experienced workplace violence in the past year. Verbal attacks were the most common type, accounting for 40.4%. More than half of all violent events (54.4%) occurred during the night shift. Of the 287 violent incidents, 58.2% occurred in the obstetrics ward, and 67.2% had other colleagues present at the time of the incident. The perpetrators of violence were mainly the family members of the patients (78.1%), and most of them were young men. Regarding to the violent incidents, half of the patients experienced obstetric violence during hospitalization (49.1%), and 48.2% of obstetric violence was directly related to the obstetric nurses who experienced WPV among the respondents. The top three characteristics of workplace violence perpetrators were low quality (65.5%), long waiting times (58.2%), and dissatisfaction with nurses’ work (53.7%). The coping styles of obstetric nurses were mainly tolerance and avoidance (77.0%), seeking help from colleagues or security guards (52.6%), and explaining patiently (52.3%). Violent incidents can lead to anger (54.0%), fear (56.1%), and reduced work quality (54.0%) among the obstetric nurses, shown in Table 2.

**Table 2:** Workplace violence (*N* = 287)

Item	Option	WPV(n(%))
Violence styles	Physical Violence	178(34.1)
	Verbal Violence	211(40.4)
	Sexual Harassment	162(31.0)
Time of event	Day shift	102(35.5)
	Middle shift	24(8.4)
	Night shift	156(54.4)
	After work	5(1.7)
At the time of the event, you were:	On duty alone	94(32.8)
	With other colleagues present	193(67.2)
Location of the incident	ward	167(58.2)
	Doctor’s Office	4(1.4)
	Nurse’s office	77(26.8)
	The corridor	28(9.8)
	Treatment Room	2(0.7)
	Other	9(3.1)
perpetrator of violence	Patient	47(16.4)
	Patient’s family	224(78.1)
	Visitor	3(1.0)
	Nurses in the department	6(2.1)
	Other	7(2.4)
Gender of the perpetrator	Male	185(64.5)
	Female	102(35.5)
The age group of the perpetrator(years)	youth(18–45)	235(81.9)
	middle-aged(46–69)	47(16.4)
	The elderly(> 69)	5(1.7)
Did “obstetric violence” occur during the hospitalization of the pregnant woman in the violent incident?	Yes	141(49.1)
	No	146(50.9)
Are you directly involved in the “obstetric violence” incident?	Yes	68(48.2)
	No	73(51.8)
Perpetrator’s situation	Patient’s death	146(50.9)
	Mental disorder	138(48.1)
	Low quality	188(65.5)
	Long waiting time	167(58.2)
	After drinking	140(48.8)
	Want to seek financial compensation	147(51.2)
	Drug abuse	135(47.0)
	Rejected unreasonable claim	137(47.7)
	Perceived high medical costs	154(53.7)
	Dissatisfaction with the doctor’s work	151(52.6)
	Dissatisfaction with nurses’ work	154(53.7)
	Unsatisfied with the treatment results	140(48.8)
	Other	137(47.7)
Coping style	Tolerate and avoid	221(77.0%)
	Explain patiently	150(52.3)
	Tit-for-tat (e.g., sparring/beating)	114(39.7)
	Reason first and fight back later	136(47.4)
	ask for help from colleagues	151(52.6)
	ask for help from security guards	151(52.6)
	Asking for help from other patients/families	133(46.3)
	Asking for help from the leader	134(46.7)
	Alarm	116(40.4)
	Other	127(44.3)
Cause of the event	Patient or family personality traits	144(50.2)
	The intensive workload of the obstetrics department	140(48.8)
	Weak interpersonal communication skills	145(50.5)
	Lack of proactive workplace violence risk assessment in the department	149(51.9)
	Maternity nurses lack awareness of the occurrence of workplace violence	149(51.9)
	Interference of adverse emotional events affecting obstetric nurses outside work	142(49.5)
	Shortage of human resources in the department	156(54.4)
	Other	131(45.6)
The impact of the event at the time	No impact	153(53.3)
	Mental inability to concentrate	153(53.3)
	aggravation	140(48.8)
	anger	155(54.0)
	Fear	161(56.1)
	insomnia	139(48.4)
	Decreased enthusiasm for work	147(51.2)
	Lower quality of work	155(54.0)
	Hate patients	142(49.5)
	Do not want to do this line	138(48.1)
	Suicidal thought	143(49.8)
	Other	140(48.8)
The impact of the incident thus far	No impact	145(50.5)
	Mental inability to concentrate	141(49.1)
	aggravation	155(54.0)
	anger	162(56.4)
	Fear	143(49.8)
	insomnia	150(52.3)
	Decreased enthusiasm for work	156(54.4)
	Lower quality of work	161(56.1)
	Hate patients	145(50.5)
	Do not want to do this line	127(44.3)
	Suicidal thought	156(54.4)
	Other	149(51.9)

Abbreviations: WPV, workplace violence

### The level of violence prevention knowledge-attitude-practice and climate perception among obstetric nurses

The mean total score of obstetric nurses’ WPV prevention knowledge-attitude-practice was 59.07 ± 15.17, and the mean score rate was 78.76% (Table 3). The average scoring rate of its subdimensions from high to low is 82.48% for the behavior dimension, 81.32% for the attitude dimension, and 72.44% for the knowledge dimension. According to the scoring rate, the overall level of obstetric nurses’ WPV prevention knowledge-attitude-practice is low (score rate < 80%), and the knowledge dimension is 72.44% with a lower score rate. The mean total score of WPV climate perception was 94.11 ± 24.69, and the average score rate was 78.43%. The average scoring rates of subdimensions from high to low are 80.26% in organizational support, 79.35% in the dimension of violence handling, 78.05% in organizational management, and 74.72% in the dimension of organizational training. According to the scoring rate, the overall level of obstetric nurses’ WPV climate perception was low (score rate < 80%), and the organizational training dimension is 74.72% with a low score rate.

**Table 3:** The level of violence prevention knowledge-attitude-practice and climate perception

Scale	Item	Minimum value	Maximum value	Mean (SD)	Score rate (%)
Violence prevention knowledge-attitude-practice	Knowledge dimension	5	25	18.11(6.03)	72.44
	Attitude dimension	5	25	20.33(5.45)	81.32
	Behavioral dimension	5	25	20.62(5.68)	82.48
	The total score	15	75	59.07(15.17)	78.76
Violence climate perception	Organizational management	4	20	15.61(4.53)	78.05
	Organizational training	5	25	18.68(6.07)	74.72
	Organizational support	7	35	28.09(7.38)	80.26
	Violence handling	8	40	31.74(9.10)	79.35
	The total score	24	120	94.11(24.69)	78.43

Abbreviations: SD, standard deviation

### The correlation between workplace violence and the level of violence prevention knowledge-attitude-practice and climate perception among obstetric nurses

The workplace violence of obstetric nurses has a significant negative correlation with the WPV prevention knowledge dimension, attitude dimension, behavior dimension, and the total score of WPV prevention knowledge-attitude-practice (Table 4). At the same time, it also has a significantly negative correlation with organizational management, organizational training, organizational support, violence handling, and the total score of WPV climate perception.

**Table 4:** The correlation between workplace violence and violence prevention knowledge-attitude-practice and climate perception

	1	2	4	5	6	7	8	9	10
Knowledge	1								
Attitude	0.669**	1							
Behavior	0.587**	0.768**							
Total score	0.858**	0.913**	1						
Organizational management	0.526**	0.639**	0.672**	1					
Organizational training,	0.461**	0.584**	0.589**	0.842**	1				
Organizational support	0.489**	0.674**	0.660**	0.803**	0.769**	1			
Violence handling	0.464**	0.588**	0.615**	0.774**	0.733**	0.753**	1		
Total score	0.527**	0.679**	0.692**	0.916**	0.900**	0.913**	0.916**	1	
Workplace violence	-0.576**	-0.602**	-0.660**	-0.581**	-0.565**	-0.553**	-0.548**	-0.613**	1

*p*, statistical significance

*p < .05; **p < .01

### Multiple linear regression analysis of workplace violence

#### Regression analysis of the level of violence prevention knowledge-attitude-practice subdimensions on workplace violence

In the workplace violence model, the knowledge, attitude, and behavior dimensions of workplace violence impact workplace violence after considering them as independent variables, which explains the 43.7% change in WPV (adjusted R^2^ = 0.437, *F* = 58.860, *P* < .001) (Table 5). From the absolute value of the regression coefficient, the influence of the three dimensions from high to low is the knowledge dimension (0.278), the attitude dimension (0.241), and the behavior dimension (0.216).

**Table 5:** Regression analysis of the level of violence prevention knowledge-attitude-practice subdimensions on workplace violence

	Unstandardized coefficient		Standardized coefficient	*t*	*p*	R^2^	Adjusted R^2^	*F*
	B	Std. Error	*β*					
constant	9.530	0.600	-	15.872	0.000**	0.445	0.437	*F*(7,514) = 58.860,*p* = .000
Age	0.106	0.158	0.040	0.671	0.503			
Years of experience in obstetric care	-0.028	0.105	-0.017	-0.267	0.790			
Professional title	0.009	0.121	0.004	0.074	0.941			
Marital status	-0.391	0.161	-0.099	-2.433	0.015*			
Knowledge dimension	-0.083	0.013	-0.278	-6.196	0.000**			
Attitude dimension	-0.080	0.019	-0.241	-4.251	0.000**			
Behavior dimension	-0.069	0.017	-0.216	-4.159	0.000**			

Dependent variable: workplace violence

Abbreviations: B, non-standardized regression coefficient; Std. Error, standard error; *β, s*tandardized coefficient; *t*, t-test coefficient; *p*, statistical significance; R^2^, decision coefficient; Adjusted R^2^, adjusted decision coefficient; *F, F*-test coefficient

*p < .05; **p < .01

#### Regression analysis of the level of violence climate perception sub-dimensions on workplace violence

The organizational management, organizational training, organizational support, and violence handling of workplace violence has an impact on workplace violence after considering them as independent variables, which can explain 38.1% of the changes in WPV (adjusted R^2^ = 0.381, *F* = 41.127, **p** < .001) (Table 6). From the absolute value of the regression coefficient, the influence of the four dimensions from high to low is organizational management (0.177), organizational training (0.175), violence handling (0.165), and organizational support (0.143).

**Table 6:** Regression analysis of the level of violence climate perception subdimensions on workplace violence

	Unstandardized coefficient		Standardized coefficient	*t*	*p*	R^2^	Adjusted R^2^	*F*
	B	Std. Error	*β*					
constant	8.55	0.621	-	13.774	0.000**	0.391	0.381	F(8,513) = 41.127, *p* = .000
Age	0.13	0.166	0.049	0.783	0.434			
Years of experience in obstetric care	-0.012	0.111	-0.007	-0.105	0.916			
Professional title	0.163	0.127	0.066	1.278	0.202			
Marital status	-0.363	0.169	-0.092	-2.15	0.032*			
Organizational management	-0.07	0.03	-0.177	-2.333	0.020*			
Organizational training,	-0.052	0.02	-0.175	-2.563	0.011*			
Organizational support	-0.035	0.015	-0.143	-2.252	0.025*			
Violence handling	-0.033	0.012	-0.165	-2.789	0.005**			

Dependent variable: workplace violence

Abbreviations: B, non-standardized regression coefficient; Std. Error, standard error; *β, s*tandardized coefficient; *t*, t-test coefficient; *p*, statistical significance; R^2^, decision coefficient; Adjusted R^2^, adjusted decision coefficient; *F, F*-test coefficient

*p < .05; **p < .01

## Discussion

The incidence of workplace violence (WPV) among obstetric nurses in this study was found to be 55.0%, slightly lower than the rate reported in the study by Pich (58%) [24]. One possible reason for this difference is that the Pich study exclusively focused on midwives, whereas our study included both midwives and ward obstetric nurses. It is well-known that the delivery room is a fast-paced environment with frequent emergencies. Moreover, the patients in this setting experience significant mood swings and severe pain, which can contribute to increased pressure and higher risks for midwives. Consequently, the incidence of WPV may be higher for midwives compared to obstetric nurses working in the ward setting. However, the study by Zhu et al. [25] did not find a higher incidence of WPV among obstetric nurses, which could be attributed to variations in the number of obstetric nurses among the respondents and the timing of the study (conducted before the implementation of the new two-child policy). Verbal aggression (73.5%) was identified as the primary type of violence in this study, consistent with the findings of the Pich study (76.7%) [24]. The high incidence of verbal violence may be attributed to the perception of many perpetrators that verbal abuse and insults are merely minor disputes with nurses, not falling within the definition of violence.

Violence by perpetrators is mainly related to their low quality, long waiting time, and dissatisfaction with nursing work, which is consistent with a previous study [26]. While the time of occurrence in this study is mainly night shifts, contrary to the previous study, it may be related to the nature of obstetric work and China’s policies under the new situation. First, as a unique specialty, obstetrics has many emergency night shifts. Under the liberalization of the childbirth policy, the number of births has soared to some extent. Second, in terms of staffing, due to the impact of the COVID-19 epidemic, various clinical departments often dispatch human resources to support, resulting in a severe shortage of the working workforce. When nurses fail to attend to the needs of patients on time, it is easy to lead to conflicts between nurses and patients and leads to WPV. More than 3/4 of the nurses tolerated and avoided treatment after the incident. The results of this study are higher than those of the study of Hemati-Esmaeili et al. in Iran [27], which may be related to the specific background and policies of each country. The reason why nurses adopt a tolerant attitude is that the reporting process is too complicated, the reporting is invalid, or the matter is irrelevant, and some nurses even feel that they will be regarded as incompetent after reporting [27–29]. Therefore, managers should simplify the reporting process, encourage nurses to report, and even adopt an incentive mechanism to improve the reporting rate of WPV. A newer finding in this study is that nearly half of the patients with violent incidents experienced obstetric violence during hospitalization (49.13%), and 47.52% had a direct relationship with obstetric nurses who had experienced WPV among the respondents. Obstetric violence will not only cause psychological harm to mothers, such as heart dissatisfaction and postpartum depression, but also affect the expected delivery of mothers and babies, such as dystocia, hemorrhage, and neonatal hypoxia [30]. At the same time, satisfaction with obstetric nursing will decrease and stimulate contradiction between nurses and patients. When the tolerance level of the mother or her family exceeds the tolerance level, they often release their negative emotions on the nurses, such as loud reprimands and roars (particularly the nurses involved in obstetric violence), leading to the occurrence of WPV. Research shows that obstetric violence is associated with nurse burnout, bonuses, and a lack of educational opportunities [31]. Managers can take steps to reduce and prevent obstetric workplace violence.

Previous research findings support the notion that workplace violence is more likely to occur among unmarried young nurses with shorter working experience and lower professional titles [24, 32–34]. Varghese’s meta-analysis highlighted that young age and limited work experience are common risk factors for workplace violence [32]. Young nurses may face challenges in communication with patients, and effective communication plays a crucial role in reducing conflicts between nurses and patients [35]. It can also help predict potential signs of workplace violence, enhance vigilance, and enable the implementation of preventive measures [36]. Nurses with shorter professional working experience may have limited proficiency in their roles. The study discovered that patient dissatisfaction with nurses’ professional abilities is a major contributing factor to hospital violence. On the other hand, nurses with rich clinical experience and professional skills are more resilient to workplace violence [37, 38]. Obstetrics, as a unique specialty, demands a high level of professional competence from its nurses, particularly in emergency response. Nurses with strong professional abilities can promptly address changes in patients’ conditions and handle various emergencies effectively. Their ability to tackle complex issues can enhance patient and family satisfaction, ultimately reducing the incidence of workplace violence. Nurses with higher professional titles often possess advanced education and extensive work experience, enabling them to better handle high-risk patients who may be prone to workplace violence [39]. In comparison, unmarried nurses, when compared to their married counterparts, may exhibit relatively weaker sense of responsibility and lack humanistic nursing experience, making them more susceptible to workplace violence.

This study showed that the overall level of obstetric nurses’ workplace violence prevention knowledge-attitude-practice is low. Each subdimension negatively correlates with the incidence of WPV, of which the knowledge dimension has the most significant impact. Only by fully understanding what WPV is and how to prevent it can WPV be stifled in the cradle, or can quick and correct responses be made when WPV occurs to avoid or reduce the negative impact of WPV. First, on the management side, relevant training should be organized regularly. Studies have shown that planned training significantly impacts nurses’ attitudes toward workplace violence [40]. Managers can distribute WPV-related materials to nurses through electronic networks and brochures. At the same time, nurses should actively acquire WPV-related knowledge, regularly participate in training, be familiar with coping plans when violence occurs, participate in simulation training, and learn from violent incidents. A previous study found that a simulation training course intervention significantly improved nurses’ self-perception and confidence in workplace violence [41]. For violence prevention, we should maintain a positive attitude. When WPV occurs, we should not be overly pessimistic, let alone over-enhanced. Studies have shown [42] that passive coping is a risk factor for WPV. Nurses with a negative attitude toward WPV are more likely to experience WPV. Many countries have introduced zero-tolerance policies and believe a zero-tolerance attitude toward WPV should be adopted. However, the latest research has found that handling depends on different situations. A zero-tolerance attitude toward physical violence is a positive coping strategy. Appropriate strategies were adopted according to the nurses’ mental resilience [43]. The author believes that before or after violence occurs, one should maintain a good attitude and state of mind, continuously increase WPV-related knowledge and coping skills, improve one’s level of violence prevention knowledge-attitude-practice, and reduce the occurrence of WPV.

This study showed that the overall level of obstetric nurses’ workplace violence climate perception was relatively low. All subdimensions were negatively correlated with the incidence of WPV, of which organizational management had the most significant impact, followed by organizational training. A previous study found that a good perception of a violent climate was conducive to improving the job security of medical staff and promoting physical and mental health [44]. To effectively reduce the incidence of WPV, experienced managers and mature management programs are needed. First, managers need to know what causes WPV. Research by Wu et al. has demonstrated that patient dissatisfaction caused by high work demands is the main reason for WPV [45]. Therefore, managers’ interventions can be implemented strategically to protect nurses from WPV and its adverse effects in managing job demands and improving the environment. Regarding management strategies, managers can use the WPV risk assessment tool for patients or visitors with violent tendencies to assess suspicious persons to identify the risk of violence, such as the STAMPEDAR assessment tool developed by Chapman et al. [46]. Establishing a safe emergency drill program and a convenient reporting process may improve nurses’ WPV prevention and reporting abilities. Meanwhile, trainers should strengthen training programs because education and training are the best measures to improve nurses’ WPV prevention and response capabilities. Gillam’s research shows that non-violent crisis intervention can reduce the incidence of WPV by conducting intensive training every six months, and the training effect can be significantly enhanced [47]. Tian’s research also suggests that managers can incorporate violence prevention into the induction training and assessment of new nurses and adopt training methods such as scenario simulation to improve the perceptions of new nurses’ violent climate [44]. It has been shown that there is little or no violence-related education in university courses [48, 49]. Nursing students need more knowledge of WPV. Tee et al. [50] proposed that nursing students should do an excellent job of training before entering clinical practice to establish WPV risk awareness, correctly understand and face WPV, which can help them prevent the occurrence of WPV in future work.

## Conclusions

Current study shows that workplace violence among Chinese obstetric nurses is relatively high in the new situation. However, violence prevention knowledge-attitude-practice and climate perception are relatively low. Workplace violence among obstetric nurses was influenced by age, years of obstetric work experience, professional title, and marital status. Nearly half of the maternal violence in hospitals is directly related to obstetric nurses who have experienced workplace violence in this study. Therefore, managers should pay attention, implement targeted interventions based on weak points, improve the perception level of obstetric nurses’ violent climate, enhance their confidence in preventing WPV, and reduce physical and mental harm. WPV-related knowledge, participating in various pieces of training, strengthening the professional skills, communication skills, and awareness of humanistic care to prevent obstetric violence will significantly improve obstetric nurses’ working atmosphere, reduce nurses’ turnover, and create an excellent occupational environment.

## Limitations

There were some limitations in this study. First, this study used a cross-sectional design and further longitudinal studies were needed to explore its causal significance. Second, although the respondents in this study came from all provinces in the country, most were in Guangdong Province. Future studies should with multi centres design including obstetric nurses from other provinces were needed.

## Implications of nursing management

This study investigated the current workplace violence among obstetric nurses in a new situation, the level of violence prevention knowledge-attitude-practice, and climate perception. It explored the correlation between violence prevention knowledge-attitude-practice and climate perception and the occurrence of WPV. The related factors fill the gap in the current literature on WPV in obstetric nurses. Our findings confirm that improving violence prevention knowledge-attitude-practice and climate perception effectively reduces workplace violence among obstetric nurses. Furthermore, WPV among obstetric nurses was strongly associated with the maternal experience of obstetric violence during hospitalization. Therefore, nursing managers can take targeted intervention measures to prevent and reduce the occurrence of WPV in obstetric nurses and promote their physical and mental health from a holistic perspective, aiming at the above-mentioned influencing factors and combining the characteristics of obstetrics.

### Electronic supplementary material

Below is the link to the electronic supplementary material.

Supplementary Material 1

## Data Availability

The datasets used or analyzed during the current study are available from the corresponding author upon reasonable request.
