# Supplementary material for: Prevalence of workplace violence in Chinese obstetric nurses under the new situation and its correlation with violence prevention knowledge-attitude-practice and climate perception: a cross-sectional study
Source: BMC Nurs. 2023 Dec 13;22:473. doi: 10.1186/s12912-023-01637-7 (PMC10717719; doi:10.1186/s12912-023-01637-7)
Supplement: Supplementary file 1 — Supplementary Material 1 [file 12912_2023_1637_MOESM1_ESM.docx]

**Obstetric nurse workplace violence questionnaire**

Dear expert,

Hello! First of all, I sincerely thank you for taking time out of your busy schedule to participate in the questionnaire survey of this study!

In recent years, with the opening of the two-child policy, the work pressure of obstetric nurses is increasing day by day, and the contradictions between patients and their families and nurses are also increasing. Workplace violence is a serious social problem widely existing in all kinds of work in the world, and it is also one of the most complex and dangerous occupational hazards in the medical and health work environment. In clinical work, obstetric nurses are often at high risk of workplace violence due to prolonged contact with patients. It is hoped that through the investigation of the occurrence of workplace violence and related factors of obstetric nurses, it can provide a basis for the formulation of prevention strategies related to workplace violence of obstetric nurses in the later stage, so as to reduce its incidence and negative impact on obstetric nurses.

**1 Instruction**

1.1 No names are recorded in this survey. The data is for the purpose of this study only and is confidential to others.

1.2 Please select the appropriate option. There are notes for multiple options.

1.3 This survey is voluntary, please fill it out according to your actual situation, thank you!

**2 Related concept description**

- 1. **workplace violence (WPV)** is defined by the World Health Organization as the exposure of health personnel to verbal abuse, threats and attacks in their workplace that cause explicit or implicit damage to their safety, well-being and health.
  2. **Obstetric violence:** Refers to violence against women during childbirth in health facilities (United Nations Committee on the Elimination of Discrimination against Women), which includes not only physical violence (such as medical treatment without informed consent, obstetric surgery, obstetric procedures and obstetric medication without evidence-based medical evidence, treatment against the patient's will), It also includes verbal violence (threats, reprimands, Shouting, belittling, lying, manipulating, mocking, etc.) and psychological violence (e.g., ignoring, condescending attitudes and expressions, contemptuous looks, impatient looks, etc.).

**3 Description of questionnaire contents**

This questionnaire consists of four parts: basic characteristics survey of obstetric nurses, workplace violence survey, violence prevention knowledge and behavior scale and violence atmosphere perception scale. The basic characteristics questionnaire contains 15 questions. Workplace Violence for Obstetric nurses includes frequency of violence (3 entries) and Incident (13 entries) sections, and if you have not experienced workplace violence, you will skip the incident section. Violence prevention knowledge and behavior contains 3 dimensions and 15 items, and violence atmosphere perception contains 4 dimensions and 24 items. Each item of the latter two scales is Likert 5-point scale (1= strongly disapprove, 5= strongly approve).

**4 Informed consent**

Do you agree to participate in this survey? (Those who agree will enter the formal questionnaire)

1. Agree B. Disagree

**Part 1: Basic characteristics of obstetric nurses**

1.Your gender is ( )

1. Male B. Female

2.Your age is ( ) years old

A.20-30  B.31-40  C.>40

3.You have been engaged in obstetric nursing for ( ) years

A.<3  B.3-5  C.6-10  D.11-20 E.>20

4. Your education background is ( )

A.Technical secondary school B. College degree

C. Bachelor's degree  D. Master's degree or above

5.Your title is ( )

A.Senior title  B. Intermediate title  C. Junior title  D. uncertain

6.Your occupation is ( )

A. Midwife B. Ward maternity nurse C. Assistant Nurse

7.Your position is: ( )

A.nurse B. Nurse group leader C. Head Nurse

8. Your current marital status is( )

A.Single  B. Married  C. Other ____

9.Are you an only child? ( )

A.Yes  B. No

10. Your form of employment is ( )

A. Formal worker  B. A contract worker  C. Temporary workers

11.Are you a local ( )

A.Yes  B. No

12.Whether you have prescriptive authority related to nursing work( )

A.Yes  B. No

13. The nature of the hospital you are currently in is ( )

A. Public General Hospital B. Public specialist hospital

C. Private General Hospital D.Private specialist hospital

14.The grade of your hospital is ( )

A.First-class hospitals B.The secondary hospital

C.Tertiary hospitals(Excluding Pre-mium Grade

15.Your present hospital is located in _______ province/municipality directly under the Central Government

**Part 2: Questionnaire on workplace violence among obstetric nurses**

**Workplace violence: Questions 1 to 15**

**1.1 The occurrence of workplace violence**

(1) In the past 12 months, someone has physically assaulted you at work (by touching you or attacking you with an object), including hitting, kicking, patting, stabbing, pushing, biting, throwing objects, twisting arms, pulling hair, etc. ( )

A. None B. Once C.2-3 times D.>3 times

(2) In the past 12 months, someone has verbally attacked you at work: (scolding, abusing, abusing, belittling or other degrading words (when: in person, by phone, by letter or by leaflet, etc.), but no physical contact ( ).

A. None B. Once C.2-3 times D.>3 times

(3)In the past 12 months, you have been sexually harassed at work: harassing language or sexual assault, such as by someone of the opposite sex

Pull, hold, kiss or touch sensitive areas ( )

A. None B. Once C.2-3 times D.>3 times

**If you have no previous experience, please skip questions 5 to 15**

**1.2 Workplace violence: Questions 5 to 15 (Please describe the most memorable incident)**

(4) Time of event:

A. Day shift B. Middle shift C. Night shift D. After work

1. At the time of the incident, you were:
2. On duty alone B.With other colleagues present
3. Place of the event:
4. ward B. Doctor's Office C. Nurse's office

D. The corridor E.Treatment Room F.Other

(7）The person who committed the violence is (if there are more than one person, only the leader is selected) :

A. patient  B. Patient's family  C. Visitor  D. Nurses in the department  E. Other ___ 

(8)The gender of the perpetrator of the violence (if there are more than one, only the leader is selected) :

A. Male  B. Female 

(9)The age of the perpetrator of the violence (if there are more than one, only the first one is selected) :

1. youth (13-17)  B. middle-aged(46-69) C. The elderly (>69 )

(10)When the incident occurred, which of the following do you think is more consistent with the situation of the abuser at that time (multiple choices can be made) : ( )

A.Patient's death 

B.Mental disorder 

C.Low quality  

D.Long waiting time

E.After drinking

F.Want to seek financial compensation

G.Drug abuse

H.Rejected unreasonable claim

I.Perceived high medical costs

J.Dissatisfaction with the doctor's work

K.Dissatisfaction with nurses' work

L.Unsatisfied with the treatment results

M.Other_____

(11)Your response to this incident (multiple options) :

A.Patient's death 

B.Explain patiently

C.Tit-for-tat (e.g., sparring/beating)  

D.Reason first and fight back later

E.ask for help from colleagues

F.ask for help from security guards

G.Asking for help from other patients/families

H.Asking for help from the leader

I.Alarm

J.Other____

(12)What do you think are the causes of this incident (multiple choices)? ( )

A.Patient or family personality traits 

B.The intensive workload of the obstetrics department

C.Weak interpersonal communication skills 

D.Lack of proactive workplace violence risk assessment in the department

E.Maternity nurses lack awareness of the occurrence of workplace violence

F.Interference of adverse emotional events affecting obstetric nurses outside work

G.Shortage of human resources in the department

H.Other____

(13)Did "obstetric violence" occur during the hospitalization of the pregnant woman in the violent incident?

A.Yes  B. No

(14)Are you directly involved in the "obstetric violence" incident?

A.Yes  B. No

(15)The impact of this incident on your psychology and behavior at that time are (multiple choices can be made) :

A.No impact 

B.Mental inability to concentrate

C.Aggravation 

D.Anger

E.Fear

F.Insomnia

G.Decreased enthusiasm for work

H.Lower quality of work

I.Hate patients

J.Do not want to do this line

K.Suicidal thought

L.Other____

(16)These psychological and behavioral effects continue to this day: (more than one) :

A.No impact 

B.Mental inability to concentrate

C.Aggravation 

D.Anger

E.Fear

F.Insomnia

G.Decreased enthusiasm for work

H.Lower quality of work

I.Hate patients

J.Do not want to do this line

K.Suicidal thought

L.Other____

**Part 3: Knowledge-attitude-practice scale of workplace violence prevention for obstetric nurses**

**Workplace violence knowledge-attitude-practice scale (according to LikerT5-level scoring method, it is divided into 5 grades: very disapprove, disapprove, general, approve, very approve, and assign 1, 2, 3, 4, and 5 points respectively)**

1. **very agree with**
2. **more agree with**
3. **generally**
4. **do not agree with**

**E.very disagree**

**(Options are shared by all questions in Part 3)**

**1.Dimensions of workplace violence prevention knowledge**

(1)I know what Workplace violence is ( )

(2)I know the manifestations of workplace violence ( )

(3)I know the causes of workplace violence ( )

(4)I know the impact of workplace violence ( )

(5)I know ways to prevent and respond to workplace violence ( )

**2.Dimensions of workplace violence prevention attitudes**

(1)I believe workplace violence can be prevented and avoided at work ( )

(2)I think workplace violence can have a serious impact on nurses, departments and hospitals ( )

(3)I think departments should establish procedures for reporting and responding to workplace violence ( )

(4)I believe that the department should regularly participate in the training and exercises related to workplace violence to improve the awareness and coping ability of nursing staff ( )

(5)I think workplace violence should be dealt with in a positive way when it happens, not just tolerated and allowed to happen ( )

**3.Workplace violence prevention behavior**

(1)I will take the initiative to learn knowledge about workplace violence, improve awareness and master coping skills ( )

(2)When I experience workplace violence, I actively report it( )

(3)I will analyze the causes of each work stress violence and make improvements to reduce the risk of workplace violence ( )

(4)I will prevent workplace violence by improving my ability to work ( )

(5)I will avoid workplace violence by improving my communication skills( ).

**Part 4: Obstetric nurses workplace violence climate perception scale**

**Workplace violence climate perception scale (according to LikerT5-level scoring method, it is divided into 5 grades: very disapprove, disapprove, general, approve, very approve, and assign 1, 2, 3, 4, and 5 points respectively)**

1. **very agree with**
2. **more agree with**
3. **generally**
4. **do not agree with**

**E.very disagree**

**(Options are shared by all questions in Part 3)**

**1.Organization management**

(1)The department provides adequate violence prevention policies ( )

(2)Department to establish a safety plan and early warning mechanism ( )

(3)The department has procedures for reporting violent incidents( )

(4)Management conducts risk assessment of violent incident cases ( )

**2.Organize training**

(5)There is regular training or continuing education on violence prevention policies and procedures within the department or hospital ( )

(6)information on the prevention of violence is distributed regularly in departments or hospitals ()

(7)The wechat public account of the department or hospital regularly has videos or articles on how to better communicate with nurses and patients and prevent violence( )

(8)In the department or hospital, nurses are trained in relevant skills to prevent the occurrence of workplace violence (such as personal communication skills, protective skills, and training to get rid of, control, and restrain violent patients). ( )

(9)Departments or hospitals regularly organize the restoration of the scene of violent incidents and simulation training to prevent problems

**3.Organizational support**

(10)Management will take appropriate measures against high-risk groups who are prone to violence (such as mental illness, substance abuse, history of violence and alcohol abuse) ( )

(11)Hospitals have institutions or organizations dedicated to dealing with "workplace violence"( )

(12)Install cameras in the work area( )

(13)Keep the night work area in the department well lit. ( )

(14)The security guards in the department set up 24-hour duty stations in the ward

(15)There are alarm buttons in the nurse's station, examination room and ward

(16)Personal protective equipment is provided for nursing staff in the department

**4.Violent treatment**

(17)The management will quickly resolve the problem of violence, which will help the nurses' mental health ( )

(18)Management takes seriously reports of workplace violence suffered by obstetric nurses( )

(19)Managers will never ignore workplace violence that damages nurses' mental health in order to improve economic performance( )

(20)Management has a "zero tolerance" policy for violence experienced by nurses at work ( )

(21)Rightly or wrongly, management protects nurses from harm first

(22)When a nurse's mental health is affected by workplace violence, management takes care of the nurse

(23)When nurses are affected by physical violence and verbal attacks on work, the management will reasonably give shift transfer or leave

(24)After nurses are subjected to violence, the management will call the nurses to analyze and summarize in time and make improvements
